# Learning to operate a high-dimensional hand via a low-dimensional controller

**DOI:** 10.3389/fbioe.2023.1139405

**Published:** 2023-05-04

**Authors:** Alexandra A. Portnova-Fahreeva, Fabio Rizzoglio, Maura Casadio, Ferdinando A. Mussa-Ivaldi, Eric Rombokas

**Affiliations:** ^1^ Department of Mechanical Engineering, Northwestern University, Evanston, IL, United States; ^2^ Department of Neuroscience, Feinberg School of Medicine, Northwestern University, Chicago, IL, United States; ^3^ Department of Informatics, Bioengineering, Robotics and Systems Engineering, University of Genoa, Genoa, Italy; ^4^ Department of Mechanical Engineering, University of Washington, Seattle, WA, United States; ^5^ Department of Electrical Engineering, University of Washington, Seattle, WA, United States

**Keywords:** dimensionality reduction, autoencoders, prosthetics, hand, learning, myoelectric, kinematics, EMG

## Abstract

Dimensionality reduction techniques have proven useful in simplifying complex hand kinematics. They may allow for a low-dimensional kinematic or myoelectric interface to be used to control a high-dimensional hand. Controlling a high-dimensional hand, however, is difficult to learn since the relationship between the low-dimensional controls and the high-dimensional system can be hard to perceive. In this manuscript, we explore how training practices that make this relationship more explicit can aid learning. We outline three studies that explore different factors which affect learning of an autoencoder-based controller, in which a user is able to operate a high-dimensional virtual hand via a low-dimensional control space. We compare computer mouse and myoelectric control as one factor contributing to learning difficulty. We also compare training paradigms in which the dimensionality of the training task matched or did not match the true dimensionality of the low-dimensional controller (both 2D). The training paradigms were a) a full-dimensional task, in which the user was unaware of the underlying controller dimensionality, b) an implicit 2D training, which allowed the user to practice on a simple 2D reaching task before attempting the full-dimensional one, without establishing an explicit connection between the two, and c) an explicit 2D training, during which the user was able to observe the relationship between their 2D movements and the higher-dimensional hand. We found that operating a myoelectric interface did not pose a big challenge to learning the low-dimensional controller and was not the main reason for the poor performance. Implicit 2D training was found to be as good, but not better, as training directly on the high-dimensional hand. What truly aided the user’s ability to learn the controller was the 2D training that established an explicit connection between the low-dimensional control space and the high-dimensional hand movements.

## 1 Introduction

With 27 degrees of freedom (DOFs) operated by 34 muscles, human hands are complex in both their structure and control. As a result, replacing the hand in cases of congenital or acquired amputation can be a challenging task, especially when the number of controlled joints is high (*e.g.,* in very dexterous prostheses) and the number of available control signals (*e.g.,* muscles) is low (Iqbal and Subramaniam, 2018). Many research groups have attempted to account for such dimensionality mismatch with existing dimensionality-reduction (DR) methods.

The most commonly used DR technique in the field of prosthetic control has been principal component analysis (PCA), which creates a low-dimensional (latent) representation of the data by finding the directions in the input space that explain the most variance in the data (Pearson, 1901). In the past, several groups have explored the efficacy of PCA in reducing dimensionality of complex hand kinematics during object grasping and manipulation (Santello et al., 1998; Todorov and Ghahramani, 2004; Ingram et al., 2008; Rombokas et al., 2012). These studies have inspired several research teams to develop a PCA-based controller for operating a prosthetic hand with multiple DOFs via a 2D (latent) space (Magenes et al., 2008; Ciocarlie and Allen, 2009; Matrone et al., 2010; Matrone et al., 2012; Segil, 2013; Segil and Controzzi, 2014; Segil, 2015).

However, PCA is purely linear in its nature, consequently only accounting for linear relationship in the input data. As a result, the nonlinear relationships that exist in hand kinematics data are disregarded. To account for these relationships, there are a variety of nonlinear DR methods. In our prior work, we have explored the use of a nonlinear autoencoder (AE) to reduce the dimensionality of hand kinematics during American Sign Language (ASL) gesturing, object grasping, and Activities of Daily Living (ADLs) (Portnova-Fahreeva et al., 2020). There, we found that two latent AE dimensions can reconstruct over 90% of the input hand kinematics data—significantly more than with PCA.

With such superior reconstruction performance, nonlinear AEs may serve as a platform for more accurate and dexterous lower-dimensional control of prosthetic hands. As a result of this work, our team has developed a myoelectric interface, in which users were able to control a virtual hand with 17 DOFs with only four electromyographic (EMG) signals (Portnova-Fahreeva et al., 2023 [*manuscript in review*]). Our preliminary work has shown the potential of AEs to be used to alleviate the issue of dimensionality mismatch in the control of dexterous prosthetic hands.

But is the dimensionality mismatch between device DOFs and control signals the only issue when it comes to myoelectric prosthetic control? Or is the problem at hand (figuratively and actually) more complex?

**FIGURE 1:**
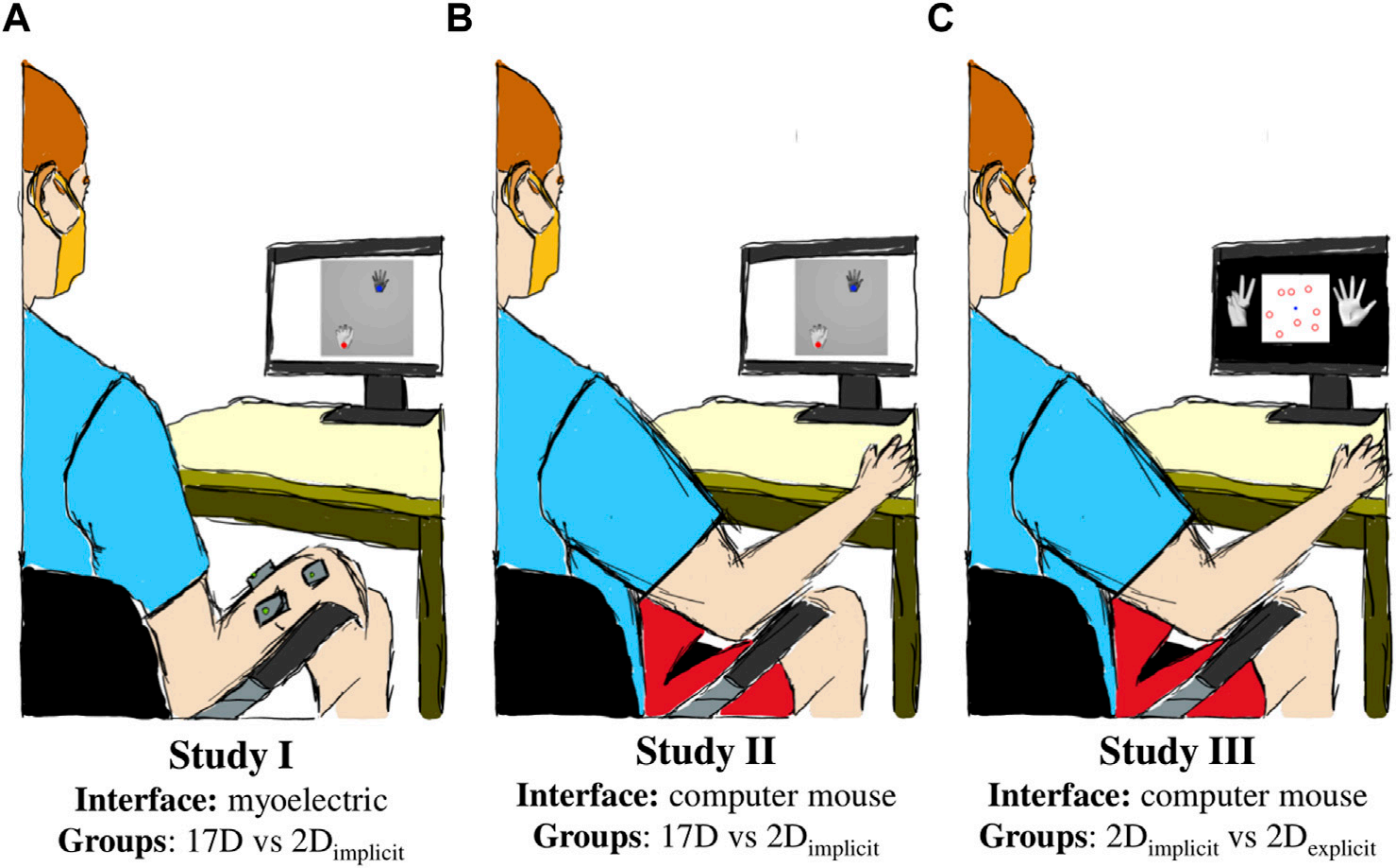
Experimental setup. **(A)** Study I used a myoelectric interface and split the participants into two groups based on training paradigms: 17D and 2D_implicit_. **(B)** Study II employed a mouse computer interface and compared two groups: 17D and 2D_implicit_. **(C)** Study III incorporated explicit target-gesture training (2D_explicit_) and a mouse-based interface.

We attempted to answer these questions with three studies in which we trained the participants to perform hand gestures on a virtual hand *via* the AE-based controller (Figure 1). The studies assessed the following things:

a) to what degree is the difficulty of operating the AE-based controller due to the complexity of operating myoelectric interfaces,
b) whether an initial training on a 2D plane, which matches the underlying dimensionality of the AE-based controller, *without explicitly* establishing the connection between 2D reaches and hand kinematics enhances learning, and
c) whether an initial training on a 2D plane, in which the user is *explicitly* told about the connection between the 2D reaches and full hand gestures, enhances learning.

Study I employed a myoelectric interface in which the participants controlled a virtual hand via the AE-based controller with by flexing/extending and abducting/adducting their wrist (Figure 1A). It explored how additional implicit training in the form of 2D target reaching affected the performance of controlling the 17D virtual hand.

Study II employed a computer mouse interface to control the virtual hand and assessed how much of the difficulty of learning the controller was from the challenge of operating an EMG-based interface (Figure 1B). Like Study I, it also assessed the potential of implicit 2D training to improve the performance of operating a 17D virtual hand.

Lastly, Study III explored how much of the difficulty in the learning arose from the user’s inability to establish the connection between the underlying 2D control and the actual 17D virtual hand (Figure 1C). Like Study II, Study III employed a mouse-based interface, but included a modified target-reaching session in 2D to establish an explicit connection for the participants between the dimensionalities of the underlying control and the presented task.

## 2 Methods

### 2.1 AE-based controller

**FIGURE 2:**
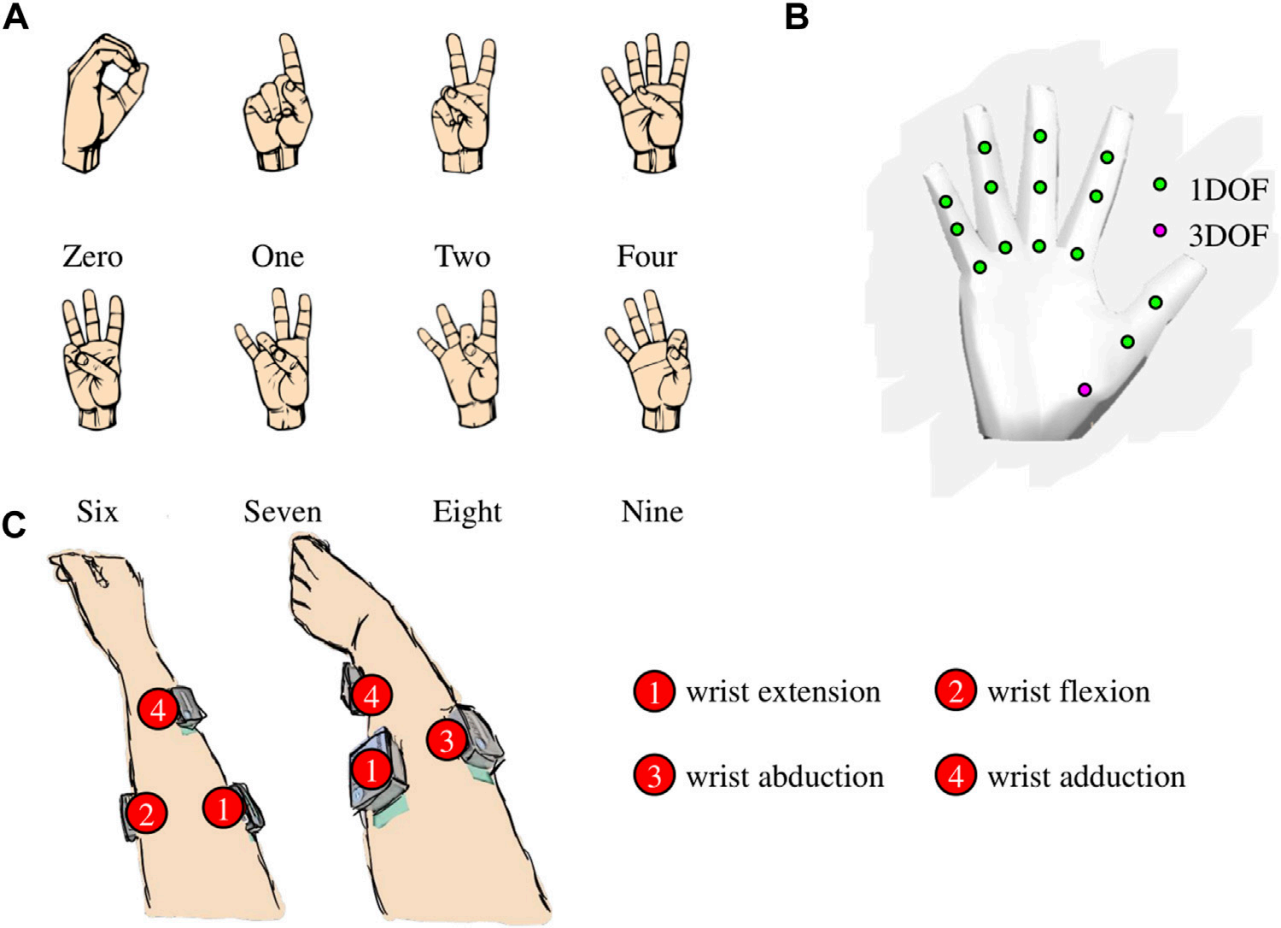
**(A)** Eight American Sign Language (ASL) gestures that the participants were trained to reproduce during the studies. **(B)** 17 degrees of a freedom (DOFs) of the virtual hand. **(C)** Surface electrode placement on the participant’s forearm for the myoelectric interface.

Using the findings of our original study, in which a nonlinear AE was determined to be superior to PCA in compressing and reconstructing complex hand kinematics, we built an AE-based myoelectric controller (Portnova-Fahreeva et al., 2023 [*manuscript in review*]). A variational AE model was used to first *encode*, or compress, high-dimensional (17D) kinematic signals recorded during ASL gestures (Figure 2A) into a low-dimensional (2D) space, and then to *decode*, or reconstruct, back into the original 17D, which corresponded to the joint angles of a virtual hand (Figure 2B).

**FIGURE 3:**
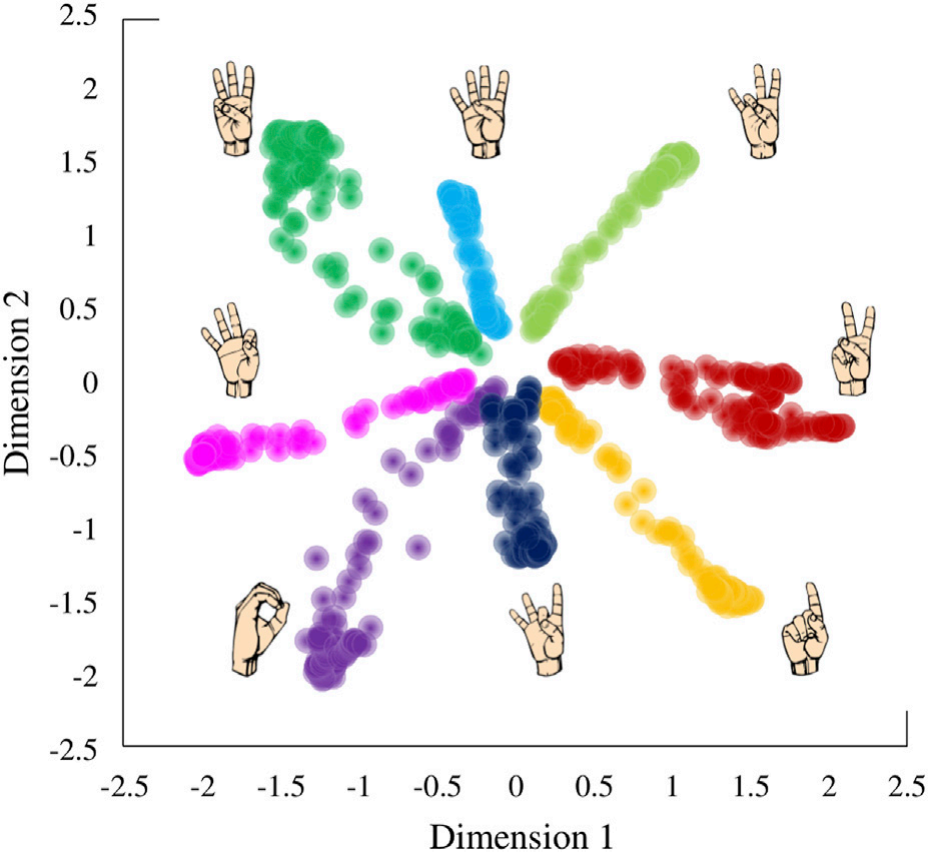
Latent space derived by applying a variational autoencoder to hand kinematics data of an individual performing American Sign Language (ASL) gestures.

This AE-based controller practically allowed the user to recreate ASL gestures in a high-dimensional hand simply by navigating along a low-dimensional 2D plane (Figure 3). Each of the points on the control space can be reconstructed into a 17D kinematic signal in a virtual hand.

#### 2.1.1 Virtual hand

The 17 DOFs of the virtual hand that were controlled were flexions/extensions of the three joints (metacarpal, proximal interphalangeal, distal interphalangeal) of the four fingers (pinky, ring, middle, and index) and flexion/extension of two joints of the thumb (metacarpal and interphalangeal) as well as the 3D rotation of its carpometacarpal joint.

Due to the nature of the AE-based control plane, not all recreated hand kinematics were within the natural ranges of motion of a biological hand. To prevent the hand from generating biologically unnatural gestures during the control, we limited the possible ranges of motion of the virtual hand joints to the ranges of motions of an actual hand. If the reconstruction output yielded a number outside of the natural range of motion of a hand joint, that joint did not change its angle in the virtual hand. For the purposes of the studies, the hand was defined to be in a *neutral gesture* when all of its fingers were fully extended (*i.e.,* open hand).

#### 2.1.2 Myoelectric interface

**FIGURE 4:**
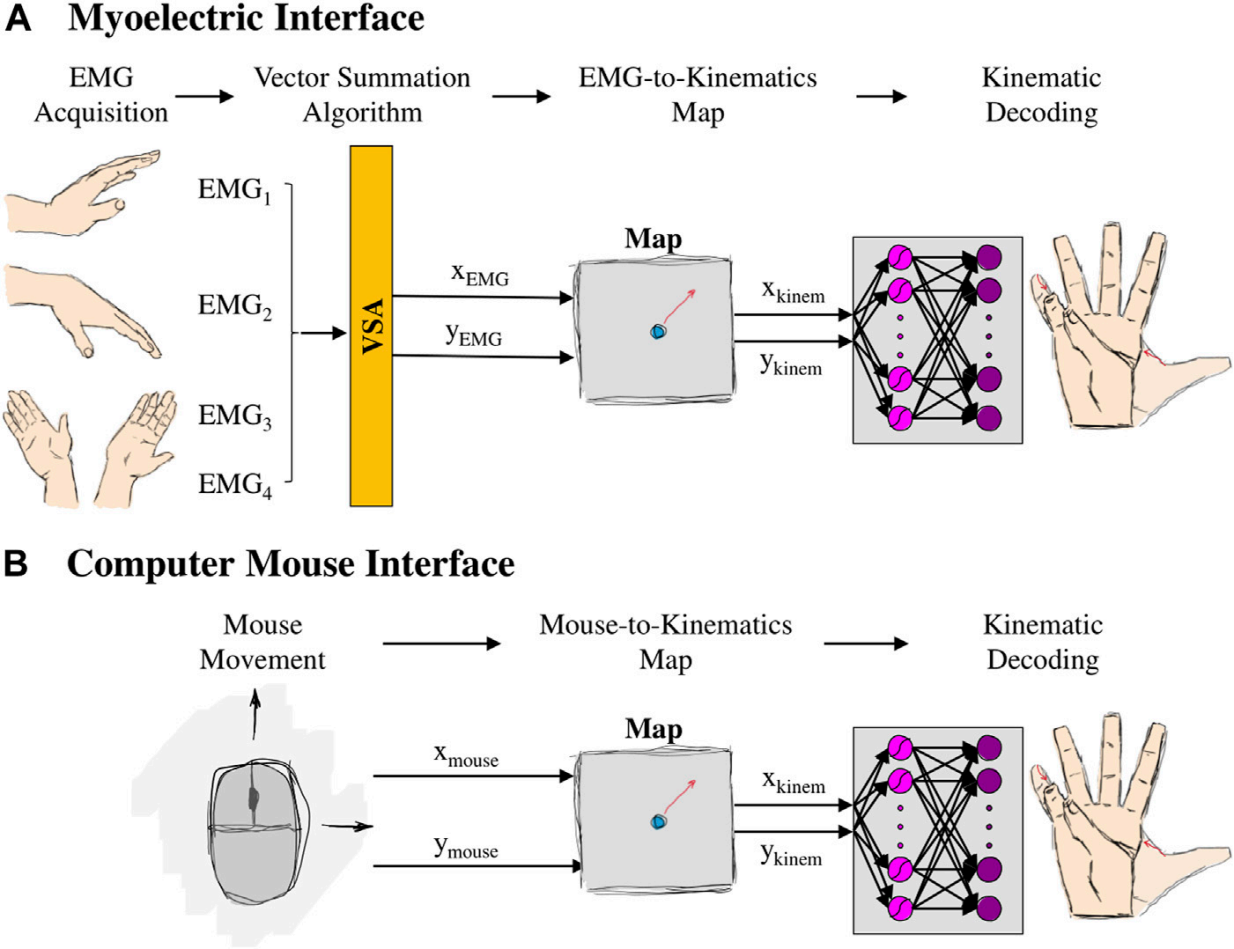
Setup of **(A)** myoelectric interface and **(B)** mouse interface. For the myoelectric interface, wrist movements generated EMG signals, which, in turn, were combined using a Vector Summation Algorithm into a 2D vector ( $X_{EMG},Y_{EMG}$ ). For the mouse interface, the 2D vector ( $X_{mouse}$ **;** $Y_{mouse}$ ) consisted of the planar position of the mouse cursor. The vector (either EMG or mouse), in turn, controlled the position of a 2D cursor on the latent space. Every point on the latent space ( $X_{kinem}$ ; $Y_{kinem}$ ) reconstructed into full 17D hand kinematics via the decoder part of the autoencoder network.

With the myoelectric AE-based controller, the user was able to operate a 17-DOF virtual hand using only four muscle signals (Figure 4A). Here is a general overview of the controller steps:

**Step 1**: four muscle signals were acquired from the user’s forearm (placement shown in Figure 2C), using surface EMG electrodes (*Delsys Inc., MA, United States*). Electrode 1 was placed approximately 1/3 of the distance between the lateral epicondyle of the elbow and the ulnar styloid process on the anterior side of the forearm. Electrode 2 was placed on the posterior side of the forearm, approximately 1/3 of the distance between the lateral epicondyle of the elbow and the ulnar styloid process, slightly more towards the ulnar side. Electrode 3 was placed on the ulnar side of the forearm, approximately 3 cm to the right of Electrode 1. Electrode 4 was placed on the anterior side of the forearm, approximately ¼ of the way between the radial styloid process and the cubital fossa. Signals from electrodes 1, 2, 3, and 4 were mainly associated with muscle activations during wrist extension, flexion, abduction, and adduction, respectively. A series of standard pre-processing techniques were applied to the raw recordings to extract the EMG envelope (*EMG Acquisition*).

To calibrate the acquired EMG signals, we used EMG recorded during 30s of rest ( $EMG_{rest}$ ) as well as structured movements ( $EMG_{struct}$ ). The structured movements consisted of seven repetitions of each of the four wrist movements (flexion, extension, abduction, adduction) at a comfortable for the participant level. For each signal i , the EMG envelope was calibrated using the maximum value recorded during rest; $\max\left(EMG_{rest,i}\right)$ , and during the structured movements; $\max\left(EMG_{struct,i}\right)$ (Pistohl et al., 2013) (Eq. 1). A scaling value, $scale_{i}$ , was also applied to ensure the participants had full coverage of the workspace without over-contracting their muscles.

$$EMG_{calib,i}=scale_{i}\frac{EMG_{i}-\max\left(EMG_{rest,i}\right)}{\max\left(EMG_{struct,i}\right)-\max\left(EMG_{rest,i}\right)}$$ (1)

**Step 2:** four calibrated EMG signals were compressed into a 2D control signal such that wrist extension/flexion controlled the vertical position ( $y_{EMG}$ ) and wrist abduction/adduction controlled the horizontal position ( $x_{EMG}$ ) (*Vector Summation Algorithm*). An offset was also added to both the x ; y directions in cases when the calibrated rest position did not appear to match the center point of the workspace (Eqs 2, 3).

$$x_{EMG}=\left(EMG_{calib,abduction}-EMG_{calib,adduction}\right)-x_{offset}$$ (2)

$$y_{EMG}=\left(EMG_{calib,extension}-EMG_{calib,flexion}\right)-y_{offset}$$ (3)

Matching the resting EMG position with the center of the latent space ensured that every trial started from the neutral gesture and every movement was performed in the center-out reaching manner. In the resting position, the corresponding virtual hand position was with all five fingers completely open.

**Step 3:** the compressed 2D control signal was mapped to the 2D control space— $x_{kinem},y_{kinem}$ , which corresponded to the cursor position on a 2D plane (Eqs 4, 5). This was done in order to account for the difference in the screen and latent space dimensions by scaling the cursor position using ${screen}_{\max}$ (*i.e.*, the length of the control plane in the local coordinate frame on the screen), and ${latent}_{\max}$ (*i.e.,* the length of the control plane in the latent space) (*EMG-to-Kinematics Map*).

$$x_{kinem}=x_{EMG}*\frac{latent_{\max}}{{screen}_{\max}}$$ (4)

$$y_{kinem}=y_{EMG}*\frac{latent_{\max}}{{screen}_{\max}}$$ (5)

**Step 4:** the point on the control space was reconstructed into 17D kinematics of the virtual hand (*Kinematic Decoding*).

More on each of these components can be found in our other work (Portnova-Fahreeva et al., 2023 [*manuscript in review*]).

Relaxing the forearm muscles returned the control position back to the center of the 2D plane, which, in turn, corresponded to the neutral gesture in the virtual hand.

#### 2.1.3 Computer mouse interface

In the computer mouse interface, the user was able to operate the virtual hand by clicking and holding the left button and dragging their computer mouse (Figure 4B). Dragging the mouse across the screen, in turn, controlled the position of the controller. Here are the steps of the mouse interface:

Step 1movement from the computer mouse on a 2D plane was obtained in the following format— $x_{mouse},y_{mouse}$ (*Mouse Movement*).

Step 2 the 2D mouse cursor position was directly mapped to the point on the control space – $x_{kinem},y_{kinem}$ (*Mouse-to-Kinematics Map*).

Step 3 the point on the control space was reconstructed into a 17D gesture of the virtual hand (*Kinematic Decoding*).

White Gaussian noise ( μ=0,σ=0.02 ) was added to the position of the controller to recreate the additive component of the neuromuscular noise of the myoelectric interface. An additional low-pass filter of 1Hz was applied to the cursor position to recreate the pre-processing delay experienced in the myoelectric interface.

Releasing the mouse button returned the control position back to the center of the 2D plane, which, in turn, corresponded to the neutral gesture in the virtual hand.

Throughout the studies, in which the mouse interface was employed, the participants did not have any visual feedback of the location of their mouse cursor. This was done in order to closely mimic the conditions of the myoelectric interface.

### 2.2 Overall protocol

For the three studies, right-handed unimpaired participants were recruited to learn to control a virtual hand on the screen via the AE-based controller. All participants were naïve to the controller. Participant recruitment and data collection conformed with the University of Washington’s Institution Review Board (IRB). Informed written consent was obtained from each participant prior to the experiment.

No physical constraints were imposed on the participants throughout the experiment as they were free to move their right arm while performing the experiment objectives.

During each study, the participants were seated in an upright position in front of a computer screen, at approximately 1.5m away at eye level. Over the span of 1 hour (for Study III) and 2 hours (for Studies I and II), they engaged in different training and test sessions to learn to recreate gestures in a virtual 17D hand via the myoelectric or mouse interface.

The gestures that they learned to recreate were eight ASL gestures (Figure 2A). Each study was divided into Training and Test phases.

### 2.3 Training phase

The training phase was divided into two sessions, referred to as *Train1* and *Train2*. The only difference between the learning groups occurred during *Train1* session of the studies.

#### 2.3.1 17D task

**FIGURE 5:**
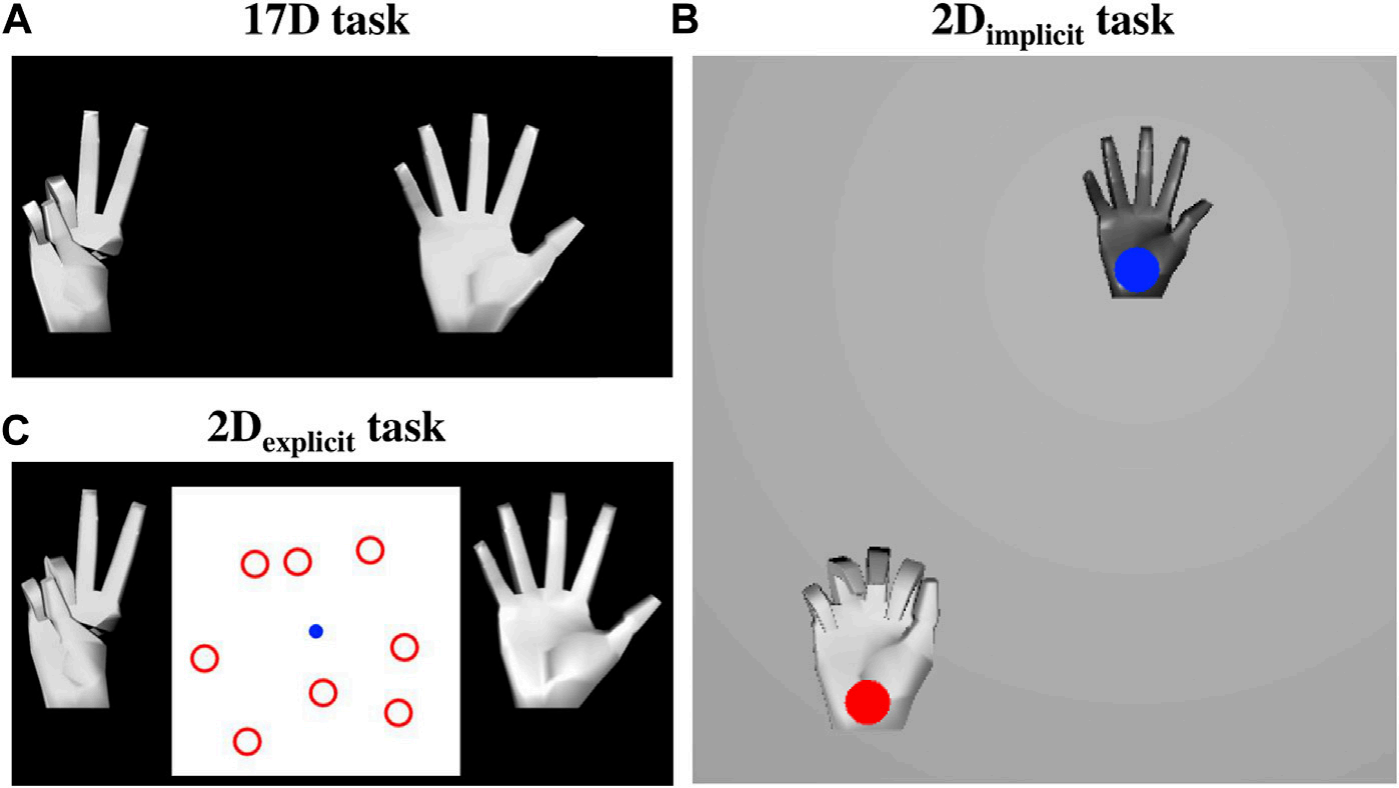
**(A)** 17D task setup. The hand on the left was the target hand that the participants needed to match. The hand on the right was the controlled hand that the participants controlled either via the myoelectric or mouse interface. **(B)** 2D_implicit_ task setup. The participants performed simple 2D reaches by controlling a hand with a blue cursor over it. The hand with the red target over it was the hand they needed to match. **(C)** 2D_explicit_ task setup. The participants controlled the blue cursor on the 2D plane, which in turn controlled the 17-DOF hand on the right. The hand on the left was the hand whose gesture they needed to match. The participants were required to learn which of the eight red targets represented the 2D location of the matching gesture.

During the 17D task, the participants were presented with only two virtual hands (Figure 5A) and had no visual feedback about the location of the controller on the 2D latent space. The hand on the left was the hand the participants needed to match. The hand on the right was the hand controlled either via a myoelectric or a mouse interface.

Each trial always started from a neutral gesture. At the beginning of a new trial, the matching hand would form a new gesture and the participants had 60s to match and hold it with the controlled hand within its acceptable range. The acceptable range in the 17D task was determined by the 2D control space. That is, if the 2D cursor related to the current hand gesture was close enough to the 2D target representing the gesture of the matching hand, then the controlled hand was within the acceptable range. The acceptable range was equivalent to 0.5 units from the center of the target on the latent space (or $0.25^{\prime \prime }$ on the screen). Once within the acceptable range, both hands turned yellow.

The participants were required to maintain the gesture for 0.75s for the trial to be counted as successful. Upon successful completion of each trial, both hands turned green.

At the end of each trial, successful or not, the participants heard a sound cue asking them to either relax their muscles (myoelectric interface) or release the button (mouse interface), which, in turn, returned the controlled hand back to the neutral gesture. Once the participant was completely relaxed for 1.5s (myoelectric interface) or released the button, a new matching gesture was presented, and a sound cue was given to start the next trial.

#### 2.3.2 2D_implicit_ task

During the 2D_implicit_ task, the participants engaged in a center-out target-reaching task. They were presented with visual feedback of the cursor that they controlled (either via a myoelectric or a mouse interface) and different targets that they needed to reach on a 2D plane (Figure 5B). The targets and the cursor were represented with circles of the same size (approximately 0.25″radius). Target locations were placed at various distances from the center cursor, which effectively were the locations of the eight ASL gestures on the latent space.

Grey and white hands were placed over both the control cursor and the target, respectively. Both hands showed the reconstructed gestures related to the current position of the cursor and the target on the latent space. The grey hand was slightly smaller in size than the white one for ease of differentiation once the two hands overlayed each other.

Each trial always started from a neutral gesture and the controlled cursor in the center of the plane. The participants were given 60s to reach the targets. If the cursor was within the acceptable range from the target, both the hands and the target turned yellow for visual feedback. The acceptable range was equivalent to 0.5 units from the center of the target on the latent space (or $0.25^{\prime \prime }$ on the screen). Once within the acceptable range, both hands and the target turned yellow.

The target was successfully reached if the cursor stayed within the acceptable range for 0.75s . Upon successful completion of each trial, the hands and the target turned green.

At the end of each trial, successful or not, the participants heard a sound cue asking them to either relax their muscles (myoelectric interface) or release the button (mouse interface), which, in turn, returned the controlled hand back to the neutral gesture. Once the participant was completely relaxed for 1.5s (myoelectric interface) or released the button, a new target was presented, and a sound cue was given to start the next trial.

#### 2.3.3 2D_explicit_ task

During their 2D_explicit_ task, the participants were always presented with both hands (the matching and the controlled ones). In addition, there was a 2D plane placed between the two virtual hands. The plane was a visual representation of the underlying control space. On the plane, there were eight red targets presented at all times, which corresponded to the 2D position of the eight gestures the participants were required to learn to recreate during training. A blue cursor corresponded to the 2D location of their controller, which, in turn, mapped into the 17D hand that the participants observed on the right (Figure 5C).

Each trial always started from a neutral gesture in the controlled hand and the blue cursor in the center of the plane. After hearing a sound cue, the hand on the left showed a new gesture, and the participants had 60s to determine which of the eight red targets produced the desired gesture in the hand on the right. Once the two hands were within an acceptable range (as described in the 2D_implicit_ task), they both turned yellow. Holding the controller within the acceptable range for 0.75s led to a successful gesture-matching, turning both hands green.

Neither the blue cursor nor the red targets provided any visual feedback on the correctness of the gesture-matching. Only the target and the controlled hands provided visual feedback by turning yellow (within the acceptable range) or green (successful) through the session. This, in turn, forced the participants to pay attention to the hand gestures as well as cursor/target location on the 2D plane, thus creating a more explicit connection between the 2D planar task and the 17D virtual hand gesture.

At the end of each trial, successful or not, the participants heard a sound cue asking them to either relax their muscles (myoelectric interface) or release the button (mouse interface), which, in turn, returned the controlled hand back to the neutral gesture. Once the participant was completely relaxed for 1.5s (myoelectric interface) or released the button, a new target and a matching gesture was presented, and a sound cue was given to start the next trial.

### 2.4 Test phase

As for the training, the test phase was also divided into two sessions, *Test1* and *Test2*. The goal of the test sessions was to determine whether the participants were able to transfer the skills acquired during training to conditions where they needed to recreate slightly different gestures. There, the participants were asked to match the hand gesture, similar to the 17D task during training either via the myoelectric or the mouse interface. No visual feedback about the location of the controller or the target gesture on the 2D plane was given during test.

**FIGURE 6:**
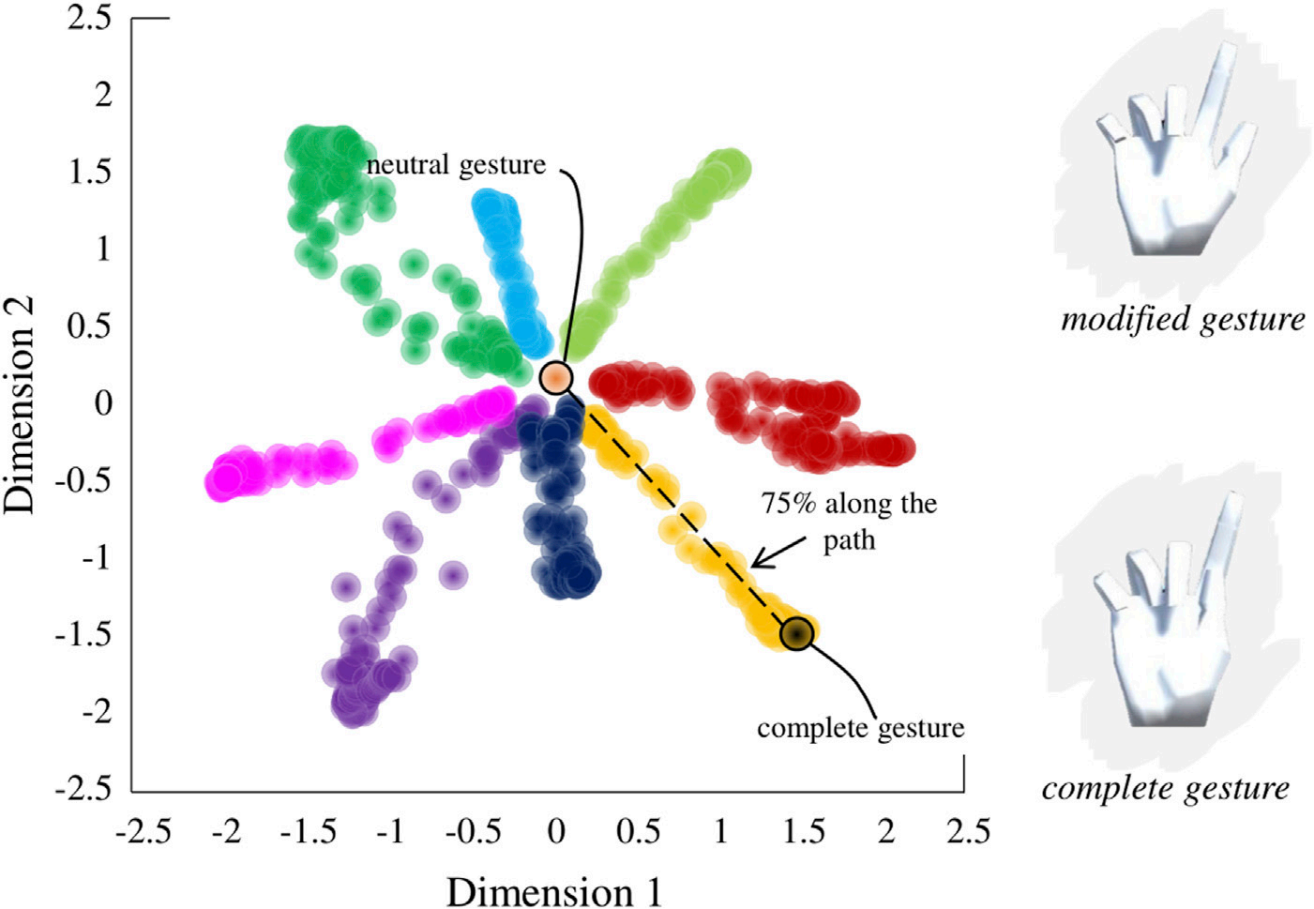
Sampling of modified gestures from the latent space. Modified gestures were sampled from 75% of the nominal path between the neutral position and the complete gesture on the latent space.

After hearing a sound cue, the participants had 6s to successfully match the gesture on the left with the hand on the right, with a 0 .75s of holding time within the acceptable range (as defined in the 2D_implicit_ task section). The gestures that they were required to match during test sessions were slight modifications of the gestures they got trained on. They were created by reconstructing a point that was 75% along the path to the gesture on the latent space (Figure 6).

As during training, both hands would turn yellow if the participant was within the acceptable range and green if they successfully matched the gesture. Each trial ended with the same cue to either relax the muscles for 1.5s (myoelectric interface) or release the button (mouse interface).

### 2.5 Participant groups

The participants in each study were split into three groups, which differed in the tasks they had to perform during *Train1*.

#### 2.5.1 Absence of 2D training (17D group)

For this group, the dimensionality of the visual feedback of the task during training did not match that of the underlying control interface. This means that the participants only performed the 17D task during *Train1*.

#### 2.5.2 Implicit 2D training (2D_implicit_ group)

For this group, *Train1* was split into the 2D_implicit_ task and the 17D task. During the 2D_implicit_ task, the dimensionality of the visual feedback of a training task matched that of the underlying control interface for part of the training. We call it *implicit* 2D training because *no explicit* explanation was given to the participants on how the target-reaching task related to the 17D task they were later presented with.

#### 2.5.3 Explicit 2D training

Similar to the 2D_implicit_ group, the 2D_explicit_ group had an initial training on the 2D control space prior to the 17D task. However, the nature of the training task (2D_explicit_ task) as well as the instructions given to the participants in this group were such that they could observe the relationship between the cursor movements on the 2D plane and the kinematics of the presented hand. In other words, this group was *explicitly* instructed on the connection between the underlying dimensionality of the controller and the generated hand gestures.

### 2.6 Studies

#### 2.6.1 Study I

For this study, we recruited 14 unimpaired right-handed individuals (four males, ten females, 25.6±5.9 years old). The participants were randomly split into two groups based on different training paradigms (17D and 2D_implicit_).

**FIGURE 7:**
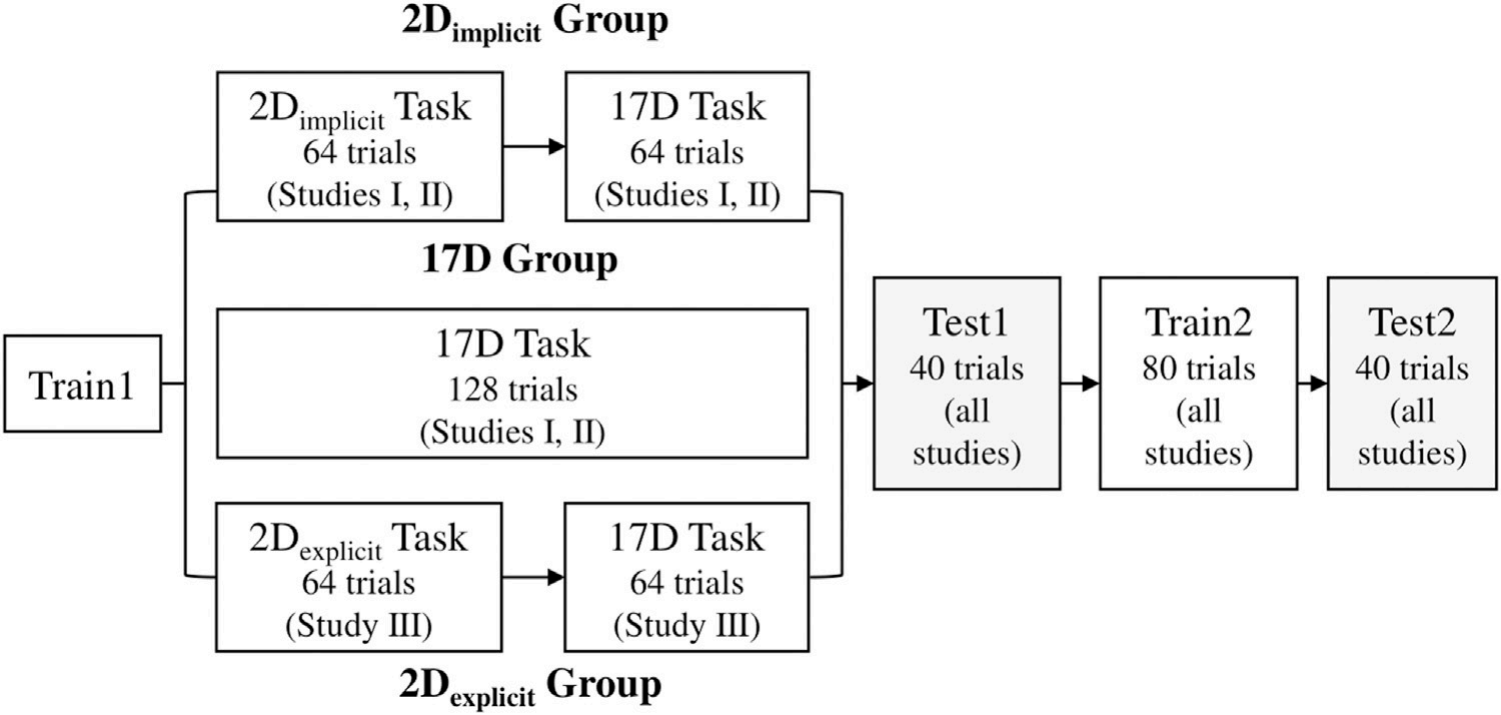
Sequence of training and test session in each study. The only difference between the groups is in *Train1* session, where the 17D group only experiences the 17D task while the 2D_implicit/explicit_ groups have the session split in half: 2D_implicit/explicit_ task and 17D task.

The main difference between the two participant groups was in *Train1*. The 2D_implicit_ group practiced the 2D_implicit_ task for the first half of *Train1* (64 trials, *i.e.,* eight gestures presented eight times in a pseudo-random order) and switched to the 17D task for the second half of the session (64 trials) (Figure 7). The 17D group performed the 17D task for the entirety of the session (128 trials, *i.e.,* eight gestures repeated 16 times in a pseudo-random order). The participants were given 1 minute to rest after every 32 trials.

During *Train2*, both groups performed the 17D task, where the eight ASL gestures were repeated 10 times in a pseudorandom order, for a total of 80 trials per session. One minute break was given to the participants after 40 trials.

During *Test1* and *Test2*, the participants were tested for a total of 40 trials during each session (*i.e.,* five modifications of eight original ASL gestures).

#### 2.6.2 Study II

A new group of 22 unimpaired right-handed participants was recruited (12 males, 10 females, 27.4±5.8 years old). All of them were naïve to the controller. As with Study I, the participants were split into two training groups: 2D_implicit_ (11 participants) and 17D (11 participants). The rest of the protocol was as in Study I.

#### 2.6.3 Study III

We recruited a total of seven participants (5 females, 2 males, 27.6±6.9 years old), who were naïve to the controller. They were all assigned to the 2D_explicit_ group operating the controller via the mouse interface (Figure 1C). There, they learned the 2D_explicit_ task for the first half of *Train1* ( 64 trials) and completed the 17D task for the second half of *Train1* ( 64 trials; Figure 7). For analysis purposes, their results were compared to 11 participants from the 2D_implicit_ group in Study II. The rest of the protocol of the protocol was as in Study I.

### 2.7 Outcome measures

Performance between and within the three groups in each study was assessed with the following metrics:

#### 2.7.1 Adjusted reach time (ART)

Adjusted reach time was defined as the time taken to complete a hand gesture match (in the 17D task) or target reach (in the 2D task). For every missed trial, the ART of the trial was set to the timeout value ( 60s ). ART was only calculated for training trials. We computed it for each training trial and then averaged the values of eight consecutive trials, which we called *repetition.*

#### 2.7.2 Adjusted path efficiency (APE)

Adjusted path efficiency was a measure of straightness of the path taken to either reach the 2D target or match the 17D gesture. It was calculated using Eq. 6, where $d_{travel}$ was the length of the path covered by the cursor to reach the target/gesture and $d_{ideal}$ was the nominal distance between the central and the final target/gesture.

$$PE=\frac{d_{ideal}}{d_{travel}}\;*100$$ (6)

Similar to ART, for every missed trial, the APE of the trial was set to the lowest possible value of 0% .

#### 2.7.3 Success rate

Success rate measured the percentage of successful trials performed in a single session. This was calculated only for the test sessions.

### 2.8 Statistical analysis

For statistical analysis, we used MATLAB Statistics Toolbox functions (*MathWorks, Natick, MA, United States*)*.* Anderson-Darling Test was used to determine the normality of the data (Anderson and Darling, 1954). Since all data were determined to be non-Gaussian, we used non-parametric tests for statistical analysis.

We evaluated differences *within* and *across* groups on the average ART and APE for the first or last repetition of the 2D_implicit/explicit_ or 17D tasks. We also calculated differences *within* and *across* compared groups for the success rate between *Test1* and *Test2* in each study. Differences *across* the groups were determined by applying Wilcoxon Rank Sum Test while differences *within* the groups were tested using Wilcoxon Sign Rank Test (Wilcoxon, 1945). In all our analyses, the threshold for significance was set to 0.05 .

## 3 Results

### 3.1 Study I

Participants across both groups were able to significantly improve their performance by the end of training (lower adjusted reach time), but no significant differences were observed across the groups in terms of final performance.

#### 3.1.1 Adjusted reach time

**FIGURE 8:**
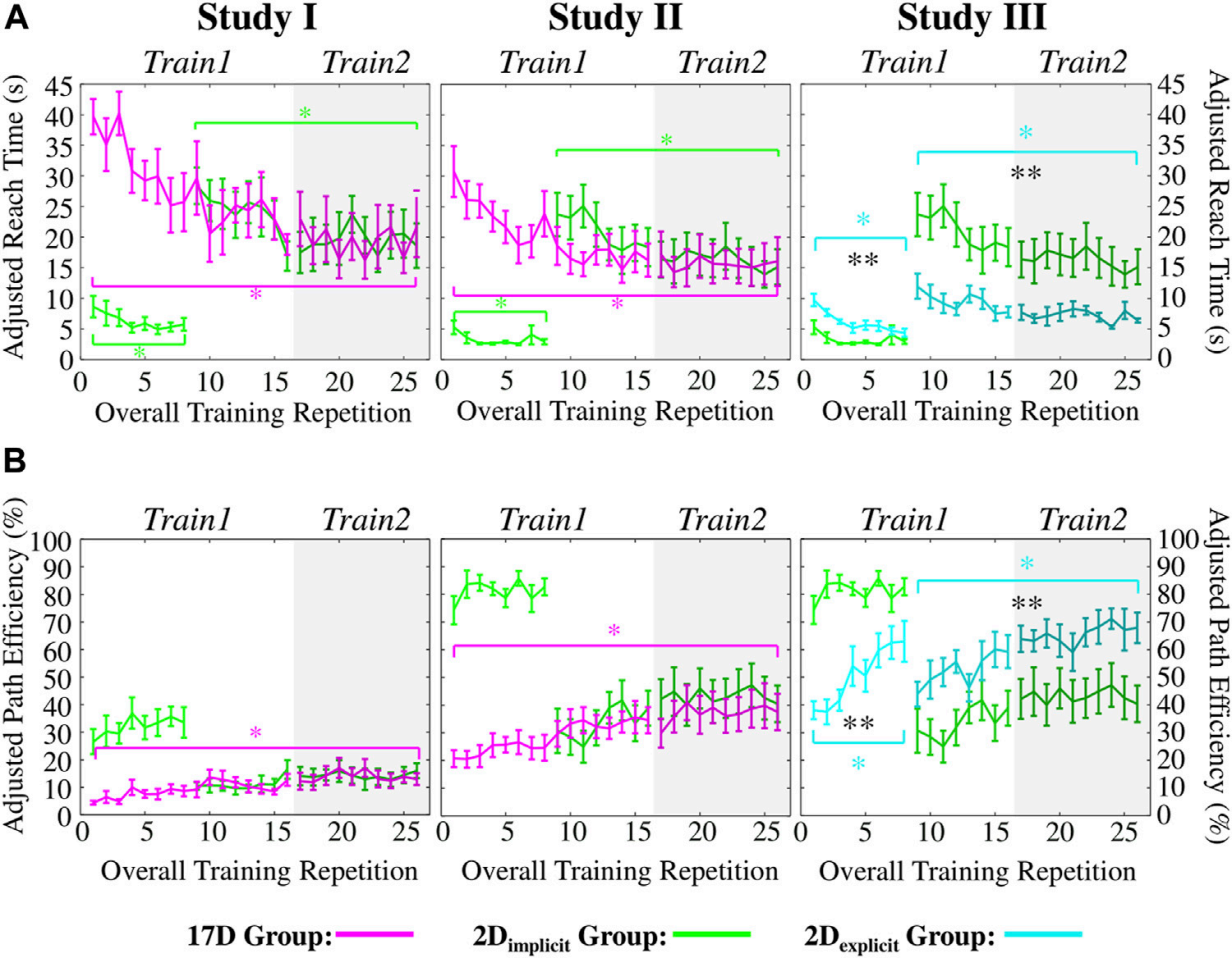
**(A)** Adjusted reach times (ARTs) and **(B)** adjusted path efficiencies (APEs) of Studies I, II, and III. Green colors represent the 2D_implicit_ group. Magenta colors represent the 17D group. Teal colors represent the 2D_exlicit_ group. Error bars represent standard errors. Magenta asterisks (*) represent statistical differences *within* the 17D group. Green asterisks (*) represent statistical differences *within* the 2D_implicit_ group. Teal asterisks (*) represent statistical differences *within* the 2D_explicit_ group. Black asterisks (*) signify statistical differences *between* groups. The shaded area of each plot represents *Train2* session. Note that for the third column (Study III), we are showing the result of the green group obtained in Study II for ease of comparison.

The 2D_implicit_ group significantly improved the ART during the 2D task—from an average of 8.6s to 5.7s ( p=0.031 ; Figure 8A *, first column*, green). It also had a significant improvement in reach time for the 17D task—ART decreased from an average of 28.4s to an average of 18.6s by the end of training ( p=0.016 ).

The 17D group also significantly decreased the ART from an average of 39.7s to an average of 21.6s by the end of training ( p=0.016 ; Figure 8A *, first column*, magenta).

The difference between the two groups at the end of the training was not statistically significant ( p=0.79 ).

#### 3.1.2 Adjusted path efficiency

The 2D_implicit_ group increased the APE from 26.5% to 33.5% during the 2D task (Figure 8B *, first column*, green). The improvement is not statistically significant ( p=0.11 ). When switching to the 17D task, the group completed the first repetition of target with an average APE of 10.5% and increased the APE to an average of 16.0% by the end of the training. Once again, the increase was not statistically significant ( p=0.08 ).

On the contrary, the 17D group was able to significantly increase its APE over the course of the 17D task training—from an average of 4.4% to 12.9% ( p=0.016 ; Figure 8B *, first column*, magenta). No differences across the groups were observed at the end of training.

#### 3.1.3 Success rate

**FIGURE 9:**
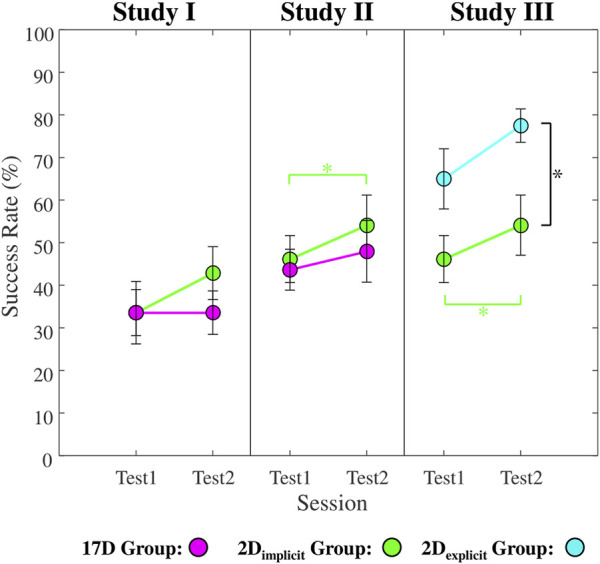
Average success rates for *Test1* and *Test2* sessions across all participants in each group in Studies I, II, and III. Green color represents the 2D_implicit_ group. Magenta color represents the 17D group. Teal color represents the 2D_explicit_ group. Error bars represent standard errors. Black asterisks (*) signify statistical differences *between* the groups in each study. Green asterisks (*) represent statistical differences *within* the 2D_implicit_ group.

The success rates of the 2D_implicit_ group during *Test1* and *Test2* were an average of 33.6% and 42.9% , respectively (Figure 9 *, first column,* green). For the 17D group, the success rates for both sessions were an average of 33.6% (Figure 9 *, first column,* magenta). Neither of the groups exhibited a statistically significant increase in the success rates during test sessions. No statistical difference was observed across the two groups.

### 3.2 Study II

Both groups were able to significantly improve their performance by the end of training (lower adjusted reach time), but no significant differences were observed across the groups in terms of final performance.

#### 3.2.1 Adjusted reach time

The participants in the 2D_implicit_ group were able to significantly improve their average ART from the first to the last repetition in the 2D_implicit_ task (from 5.3s to 2.9s , respectively) ( p<0.01 ; Figure 8A *, second column,* green). The group was able to significantly improve its ART for the 17D task as well—from 23 .6s for the first repetition and 15.1s for the last repetition during training ( p<0.01 ).

Similarly, the 17D group had a significant improvement in its average ART value—from 30 .7s to 16.0s ( p<0.01 ; Figure 8A *, second column*, magenta).

The difference between the two groups at the end of the training was not statistically significant ( p=0.79 ).

#### 3.2.2 Adjusted path efficiency

The increase of the APE values for the 2D_implicit_ group during the 2D_implicit_ task was not statistically significant ( p=0.37 ): from 74.3% to 82.7% (Figure 8B, green). Similarly, the increase in the APE during the 17D task was from 30.7% to 40.0% and not statistically significant ( p=0.10 ).

As in Study I, only the 17D group significantly increased its average APE value over the course of the training repetitions—from 20.7% to 37.5% ( p=0.024 ; Figure 8B, magenta). Similar to Study I, no differences across the groups were observed at the end of training.

#### 3.2.3 Success rate

During test sessions, the 2D_implicit_ group successfully completed 46.1% of *Test1* and 54.1% of *Test2* (Figure 9 *, second column,* green). The difference between the test session was statistically significant ( p=0.037 ). For the 17D group, *Test1* and *Test2* were successfully completed at a rate of 43.6% and 48.0% , respectively (Figure 9 *, second column,* magenta), although the improvement was not statistically significant ( p=0.38 ). No statistical difference was observed across the two groups in neither of the test sessions.

### 3.3 Study III

In the sections below, we compare the performance of the 2D_implicit_ group from Study II and the 2D_explicit_ group, whose data were collected during Study III. In addition to significantly improving their performance over the entire training, participants in the 2D_explicit_ group significant outperformed the 2D_implicit_ group in terms of reach time and path efficiency.

#### 3.3.1 Adjusted reach time

The participants in the 2D_explicit_ group significantly decreased their average ART during the 2D_explicit_ task—from 9.7s to 4.4s ( p=0.047 ; Figure 8A, *third column,* teal). Once the visual presentation of the targets, cursor, and the 2D plane was switched off, the participants completed the first repetition of the 17D task within 12.0s on average and were able to significantly reduce the ART to at an average of 6.4s by the end of the training ( p=0.031 ).

During the 2D_implicit/explicit_ tasks alone, the 2D_implicit_ group reached targets significantly faster than the 2D_explicit_ group at the beginning ( p=0.01 ) and the end of the 2D task trials ( p<0.01 ).

However, the 2D_explicit_ group began the 17D task at a significantly lower ART value ( 12.0s ) than the 2D_implicit_ group ( 23.7s ) ( p=0.02 ). At the end of the training, the 2D_explicit_ group was also successfully matching gestures at a significantly faster ART ( 6.4s ) than the 2D_implicit_ group ( 15.1s ) ( p<0.01 ).

#### 3.3.2 Adjusted path efficiency

The 2D_explicit_ group significantly improved its APE during both the 2D_explicit_ and the 17D tasks (Figure 8B *, third column,* teal). During the 2D_explicit_ task, the improvement was from an average of 38.1% to 62.9% ( p=0.03 ). During the 17D task, the improvement was from an average of 43.9% to 67.9% ( p=0.02 ). In addition, the final APE of the 2D_explicit_ group at the end of the 17D task ( 67.9% ) was similar to the level that the group was able to achieve by the end of the 2D_explicit_ task ( 62.9% ) ( p=0.38 ).

When considering the 2D_explicit/implicit_ tasks, the 2D_excplit_ group had consistently lower APE values than the 2D_implicit_ group. When switching to the 17D task, the difference of average APE values between the 2D_explicit_ ( 43.9% ) and 2D_implicit_ ( 30.7% ) groups was not statistically significant ( p=0.13 ). However, by the end of the training, the 2D_explicit_ group was able to perform significantly more efficient reaches ( 67.9% ) than the 2D_implicit_ group ( 40.0% ) ( p=0.01 ).

#### 3.3.3 Success rate

The success rate of the 2D_explicit_ group between the test sessions improved from an average of 65% to an average of 77.5% (Figure 9 *, third column*), although not significantly ( p=0.078 ). In addition, when comparing the two groups, the average success rate during *Test2* was significantly higher for 2D_explicit_ group ( 77.5% ) than the 2D_implicit_ one ( 54.1% ) ( p=0.02 ).

## 4 Discussion

The set of studies presented in this paper allowed us to answer the three questions proposed in the beginning of the paper:

a) To what degree is the difficulty of operating the AE-based controller due to the complexity of operating myoelectric interfaces?

The complexity of the myoelectric interface did not have an effect on the final performance in terms of reach times achieved by either of the tested groups—the participants were able to perform gesture matches as fast as their counterparts who used the mouse interface. The only difference was observed in terms of path efficiency—the participants using the mouse interface were able to perform straighter reaches than the participants using the myoelectric interface.

b) Does an initial training on a 2D plane, which matches the underlying dimensionality of the AE-based controller *without explicitly* establishing the connection between 2D reaches and hand kinematics, enhances learning?

Without an explicit connection between the 2D reaches and virtual hand kinematics, the participants practicing the 2D_implicit_ task did as well, but not better than their counterparts who practiced only the 17D task.

c) Does an initial training on a 2D plane, in which the user is *explicitly* told about the connection between the 2D reaches and full hand gestures, enhances learning?

Providing an explicit connection between the underlying low-dimensional control space and the presented high-dimensional task significantly improved the participant’s performance in terms of reach times and path efficiencies.

### 4.1 Difficulty of myoelectric control

The findings in Studies I and II suggest that the myoelectric interface itself was not the main reason for poor performances across Study I participants and highlighted the need to look further into more optimal ways of teaching the users about the controller itself to improve their overall performance.

The notion that the users can learn any mappings for human-computer interfaces that you provide them with (intuitive or not) has been supported by a multitude of studies (Liu and Scheidt, 2008; Radhakrishnan et al., 2008; Ison and Artemiadis, 2015; Zhou et al., 2019; Dyson et al., 2020). However, we hypothesized that learning the control map may be more difficult in cases when a developed controller is operated via input signals in an unnatural way. What we mean by that is, for example, in case of our myoelectric interface, the forearm muscle contractions (*i.e.,* wrist movements) did not yield the same physiological kinematic results in the virtual hand. As a result, there was a clear mismatch between the natural way of creating the gestures the users saw on the screen (*i.e.,* finger flexions and extensions) and the alternative way they were required to learn to use their wrist to recreate these gestures. For comparative purposes, we utilized a computer mouse interface, which, we hypothesized, would allow the users to learn the mapping significantly faster, given than it is more familiar and used every day.

**FIGURE 10:**
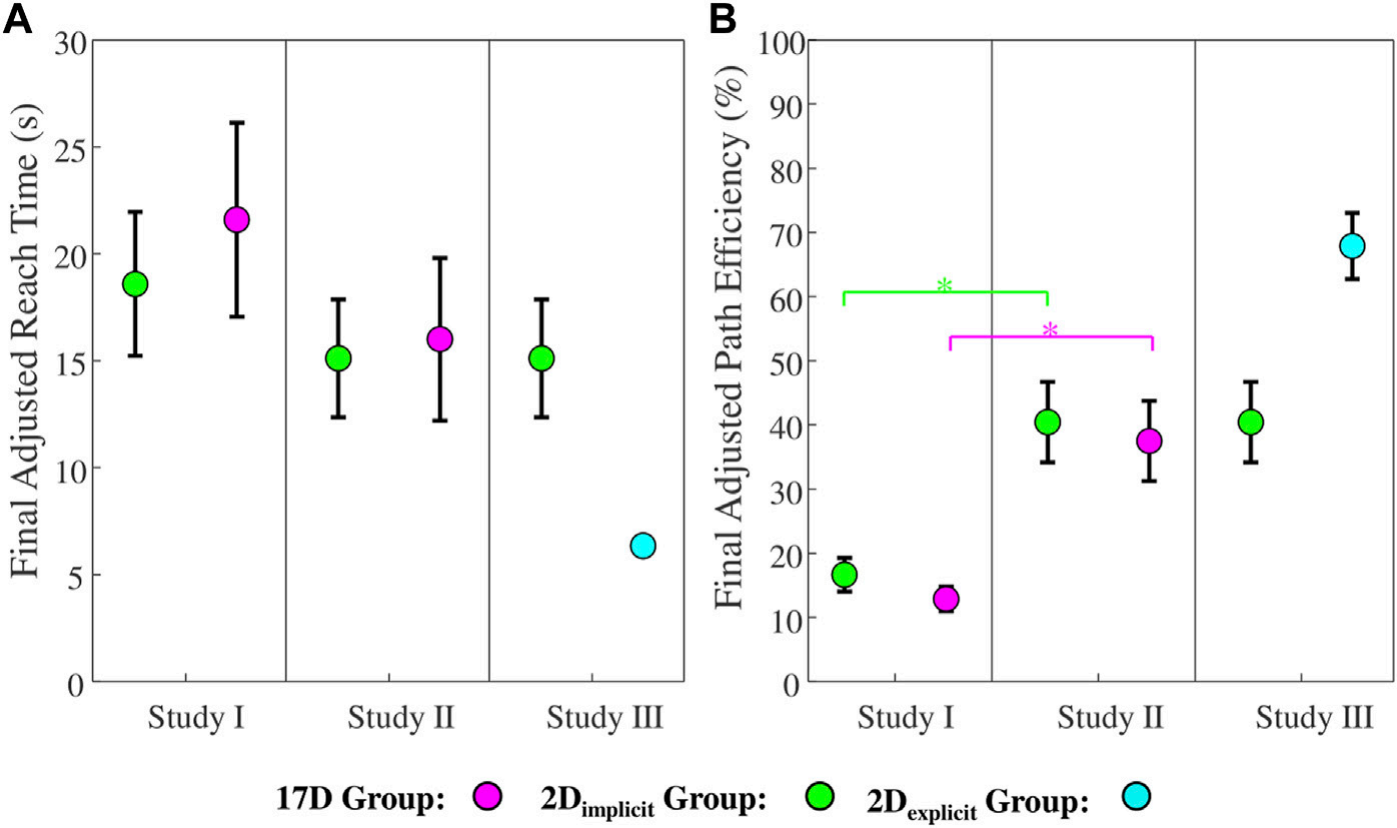
**(A)** Adjusted reach time and **(B)** adjusted path efficiency for the final training repetition across the 2D_implicit_ (green), 17D (magenta), and 2D_explicit_ (teal) groups in Studies I, II, and III. Green asterisks (*) represent statistical differences between the 2D_implicit_ groups in Studies I and II. Magenta asterisks (*) represent statistical differences between the 17D groups in Studies I and II.

Despite our hypothesis, we found that by the end of the training, there was no significant difference in the reach times between the participants in Studies I and II ( p=0.43 for 2D_implicit_; p=0.25 for 17D; Figure 10A). The only main significant difference between Studies I and II was that by the end of the training, the participants who used a computer mouse were able to match gestures in a significantly more efficient way (higher adjusted path efficiency) than the participants who operated the myoelectric interface ( p=0.01 for 2D_implicit_; p=0.01 for 17D; Figure 10B). This observation appears to be self-evident—people perform straighter reaches by moving the mouse with their hand rather than trying to activate their muscles. In addition, it is important to note that myoelectric signals are noisier than the ones produced by moving a mouse, hence straighter trajectories with the mouse interface are expected. Lastly, we can observe that following *Train2*, both groups in the mouse interface completed *Test2* session at significantly higher success rates than their myoelectric counterparts ( p= for 2D_implicit_; p= for 17D; Figure 9), suggesting that the myoelectric controller presented additional challenges that slowed down learning.

It is also important to note that control gains across the two interfaces were potentially not the same, thus, making a direct comparison between Studies I and II in terms of reach times difficult. In the case of a myoelectric interface, control gains were individualized to each participant to ensure that they could access each point on the 2D control plane with comfortable muscle contraction levels (*i.e.,* without over-contracting). On the contrary, in the mouse interface case, the participants had fixed control gains. It is possible to assume that the difference in the initial reach times during *Train1* in the 17D group between the myoelectric and mouse interfaces was due to the control gains for the mouse controller being larger. However, when observing the final reach times in *Train2*, participants across both Study I and Study II plateaued to comparable reach times for both interface types, suggesting that control gains were potentially comparable.

### 4.2 Effects of implicit and explicit training

In Study III, we found that guiding the user to learn the explicit connection between the underlying dimensionality of the controller and the high-dimensional hand postures was critical for learning.

#### 4.2.1 2D_implicit_ training

Here, the relationship between the underlying dimensionality of the controller and the gesture-matching task that followed the target reaching was not explicitly made. Presentations of a single target at a time along with smaller avatars of the controlled and matching hands, most likely, encouraged the participants to focus on the 2D target location rather than the generated 17D gesture. This observation is supported by the fact that the 2D_implicit_ group did not outperform the 17D group during gesture-matching at the end of *Train2* (Studies I and II).

It is also important to note that after the first eight repetitions of either the 17D or 2D_implicit_ task, both groups completed gesture matching at comparable adjusted reach times (Figure 8A). This points to the fact that the initial training in 2D worked as well, but not better, as having the initial training on the 17D task. The same effect is observed when looking at path efficiency during Studies I and II—the participants across both groups performed similarly (Figure 8B).

#### 4.2.2 2D_explicit_ training

When designing Study III, we hypothesized that learning that took place during the 2D task did not provide the 2D_implicit_ group with a full understanding of the underlying dimensionality of the control space. Instead, it only trained them to perform abstract (as they appeared to the participants) movements on a 2D plane. As a result of this observation, in Study III we explicitly informed the participants about the relationship between the cursor position in 2D and the corresponding (i.e., reconstructed) hand gesture in 17D. We hypothesized that once this relationship was understood, it would become a pure memorization problem of the gesture locations on the 2D plane.

The presentation of all eight targets without error feedback directly on the targets themselves forced the participants to pay attention not only to the target locations but at the desired hand gestures. In addition, the participants clearly observed how their movements on the 2D plane corelated to the generated gestures as the hands were now more visually accentuated and significantly larger than those presented in Studies I and II.

### 4.3 Linear vs. nonlinear postural controllers

We hypothesize that the explicit understanding of the controller-task relationship would be essential when teaching nonlinear-based controllers developed; however, such understanding might not be relevant in cases where PCA (a *linear* method) has been used. The reason for that would be that the latent space created by PCA follows the superposition principle. This means that new gestures could be superimposed from other gestures on the 2D plane. A good example of that would be if one dimension of the latent space solely controls the opening and closing of the thumb, while the other one flexes and extends the other four fingers, gestures generated within the space would be linear combinations of these two dimensions.

The superposition principle does not hold in latent spaces generated by nonlinear systems such as nonlinear AEs. And while gestures that are similar kinematically, appear closer to each other on the latent space encoded by a nonlinear AE (Portnova-Fahreeva et al., 2020), nonlinear maps might be harder for users to interpolate from. And while the differences in learning linear and nonlinear maps have not been explored in this paper, we suggest that this might be an interesting route to investigate in future experiments.

One of the major outcomes of the studies described in this paper was the application of nonlinear DR methods, such as AEs, for the development of a *nonlinear* postural controller, in which complex kinematics of a virtual hand with 17DOFs were extracted from the position on the 2D plane. In the past, *linear* controllers have been developed, in which the dimensionality of hand kinematics during grasping was reduced using PCA (Magenes et al., 2008; Matrone et al., 2010; Matrone et al., 2012; Belter et al., 2013; Segil, 2013; Segil and Controzzi, 2014; Segil, 2015).

In the studies where linear postural controller was validated via a myoelectric interface (Matrone et al., 2012; Segil and Controzzi, 2014; Segil, 2015), the average movement times (time to successfully reach but not hold the hand in a correct grasp) were between 3s and 5s . The results are comparable to those produced in our Study III; however, they differ in the interface used to perform the movement (our Study III employed the mouse interface). When compared to the results of our myoelectric interface study (Study I), movement times using the PCA-based controller were significantly lower than those using the AE-based controller.

Explanations for the discrepancies in the results could be due to the major differences in the controller schemes and protocols. First of all, the output system with a PCA-based controller had 5−6 DOFs, in contrast to the 17 DOFs controlled in our studies, resulting in a more intricate but, most likely, complex control. In addition, in one of the aforementioned studies (Matrone et al., 2012), the participants were only required to learn to create three grasps in comparison to learning eight unique ASL gestures in our studies. Another study (Segil, 2015) implemented potential fields that “snapped” the virtual hand in correct postures when the control cursor was close enough to the target posture on the 2D plane. That allowed for simpler control and alleviated the challenges experienced during myoelectric control due to the noisy nature of EMGs.

Despite potential differences across the linear- and nonlinear-based controllers, the nonlinear counterpart yielded a major advantage in its superiority in reconstructing higher variance of the input signal with a smaller number of latent dimensions (Portnova-Fahreeva et al., 2020). What this means is that a nonlinear-based controller with just two latent dimensions would result in a reconstructed hand that was closest in appearance (*i.e.,* kinematically) to the original input signals whereas the PCA-based controller would be unable to reconstruct some of the gestures.

Following the results of Study III, in which we discovered a more effective form of training of the AE-based controller via the mouse interface, we hypothesize that a higher performance than what was observed in Study I could be achieved with a nonlinear postural controller via a myoelectric interface. As a result, we suggest that nonlinear postural controllers could still be a viable option for complex prosthetic control allowing for more precise dimensionality-reduction of intricate hand kinematics than what could be achieved by PCA.

### 4.4 Limitations

One of the major limitations of our studies was the design of the test sessions with very short window to perform reaches. Considering that the average ART during training sessions in Studies I and II was significantly higher than the time allowed for a successful reach during test, the participants were set up for failure, which explains the low success rates during test. In Study III, the average ART at the end of the training sessions was similar to the time allowed for a successful gesture-matching during test, which explains a significantly higher success rates during test sessions for the 2D_explicit_ group.

As discussed in Section 4.1, given the experimental design, the control gains between the mouse and myoelectric interfaces across the studies are not directly comparable. To allow the participants in Study I to be able to reach every point on the 2D control plane without over-contracting their muscles, we tuned the controller gains for each individual. The control gains in the mouse interface studies (Studies II and III), on the contrary, were kept constant for all participants. As a result, this design decision might have had an impact on the comparability of the results across myoelectric and mouse-controlled studies. In addition, this makes it difficult to assume that the 2D_explicit_ group results from Study III (in terms of faster reach times) would translate entirely to a setup with the myoelectric interface. However, given that adjusted reach times plateaued around similar values for the groups training with the mouse interface in Study II as the groups training with the myoelectric interface in Study I (Figure 8A), it is possible that a 2D_explicit_ group would get to similar reach times by the end of training using the myoelectric interface as the same group did using the mouse interface in Study III.

At the surface level, myoelectric noise can be modeled as Gaussian noise with both additive and multiplicative components (Clancy et al., 2001). Since the level of muscle activation required to reach the entire workspace in Study I was kept at comfortable (non-over-contracting) levels by design, we assumed that the multiplicative component would likely be similar to its additive counterpart, thus resulting in the design only incorporating the additive component. However, this assumption, if false, might have resulted in the mouse interface not being as noisy as the myoelectric interface, thus making direct comparisons between the path efficiency results between Studies I and II more difficult.

Lastly, the fastest reach times presented by the 2D_explicit_ group by the end of training in Study III (an average of 6.4s ) are still long for any action, especially for prosthetic control. While this work only investigated various aspects that improve or inhibit learning of the novel nonlinear AE-based controller, other solutions can be explored that improve reach times. For example, control gains could be increased if a stable performance was achieved or “potential fields” such as those implemented by Segil and others (Segil, 2015) to allow for the controller to “snap” into specific gestures once the user is near it on the 2D control plane.

### 4.5 Applicability for prosthetic users

When designing these studies, the end-user group that we considered were upper-limb amputees that utilize prosthetic hands in their daily living. Although the studies were performed on unimpaired individuals, they highlighted the possibility of using nonlinear controllers for the purpose of manipulating a myoelectric hand prosthesis. The myoelectric interface that we designed for Study I employed wrist muscle signals to operate on the 2D latent space. And although an upper-limb amputee might not have those wrist muscles, other more proximal locations can be chosen to obtain clean signals to control a location of a 2D cursor, which, in turn, would operate the hand. The main advantage of our controller is that it does not require a large number of signals (only enough to operate the cursor on the 2D plane) to control a hand with a large number of DOFs. One does not even need to limit themselves to the EMG system. One suggestion would be to obtain a 2D control signal from a simpler interface based on Internal Measurement Units (IMUs). For example, IMUs can be placed on the user’s shoulders, consequently, controlling the posture of the prosthetic hand. In the past, IMUs have been widely used to operate a low-dimensional controller (Thorp et al., 2015; Seáñez-González et al., 2016; Abdollahi et al., 2017; Pierella et al., 2017; Rizzoglio et al., 2020). Thus, nonlinear AE-based controllers, such as the one we developed for our studies, can be a versatile and modular solution for controlling complex upper-limb prosthetic devices via low-dimensional interfaces.

## Data Availability

The raw data supporting the conclusions of this article will be made available by the authors, without undue reservation.
